# A Novel Scoring System to Measure Radiographic Abnormalities and Related Spirometric Values in Cured Pulmonary Tuberculosis 

**DOI:** 10.1371/journal.pone.0078926

**Published:** 2013-11-01

**Authors:** Renata Báez-Saldaña, Yesenia López-Arteaga, Alma Bizarrón-Muro, Elizabeth Ferreira-Guerrero, Leticia Ferreyra-Reyes, Guadalupe Delgado-Sánchez, Luis Pablo Cruz-Hervert, Norma Mongua-Rodríguez, Lourdes García-García

**Affiliations:** 1 Instituto Nacional de Enfermedades Respiratorias (INER), Mexico, D.F., Mexico; 2 Instituto Nacional de Salud Pública (INSP), Cuernavaca, Morelos, Mexico; McGill University, Canada

## Abstract

**Background:**

Despite chemotherapy, patients with cured pulmonary tuberculosis may result in lung functional impairment.

**Objective:**

To evaluate a novel scoring system based on the degree of radiographic abnormalities and related spirometric values in patients with cured pulmonary tuberculosis.

**Methods:**

One hundred and twenty seven patients with cured pulmonary tuberculosis were prospectively enrolled in a referral hospital specializing in respiratory diseases. Spirometry was performed and the extent of radiographic abnormalities was evaluated twice by each of two readers to generate a novel quantitative score. Scoring reproducibility was analyzed by the intra-class correlation coefficient (ICC) and the Bland-Altman method. Multiple linear regression models were performed to assess the association of the extent of radiographic abnormalities with spirometric values.

**Results:**

The intra-observer agreement for scoring of radiographic abnormalities (SRA) showed an ICC of 0.81 (CI:95%, 0.67–0.95) and 0.78 (CI:95%, 0.65–0.92), for reader 1 and 2, respectively. Inter-observer reproducibility for the first measurement was 0.83 (CI:95%, 0.71–0.95), and for the second measurement was 0.74 (CI:95%, 0.58–0.90). The Bland-Altman analysis of the intra-observer agreement showed a mean bias of 0.87% and -0.55% and an inter-observer agreement of -0.35% and -1.78%, indicating a minor average systematic variability.

After adjustment for age, gender, height, smoking status, pack-years of smoking, and degree of dyspnea, the scoring degree of radiographic abnormalities was significantly and negatively associated with absolute and percent predicted values of FVC: -0.07 (CI:95%, -0.01 to -0.04); -2.48 (CI:95%, -3.45 to -1.50); and FEV_1_ -0.07 (CI:95%, -0.10 to -0.05); -2.92 (CI:95%, -3.87 to -1.97) respectively, in the patients studied.

**Conclusion:**

The extent of radiographic abnormalities, as evaluated through our novel scoring system, was inversely associated with spirometric values, and exhibited good reliability and reproducibility. As intra-observer and inter-observer agreement of the SRA varied from good to excellent, the use of SRA in this setting appears acceptable.

## Introduction

Globally, tuberculosis remains a significant cause of high morbidity and mortality, with 1.4 million deaths and 8.7 million new cases recorded in 2011. Tuberculosis is also a significant health issue in Mexico, where an estimated minimum of 18 persons per 100,000 inhabitants develop tuberculosis each year [1]. In 2011, 15,843 new cases of pulmonary tuberculosis were identified, representing 81.6% of all cases of tuberculosis recorded that year [2]. The pathologic hallmark of tuberculosis is the formation of granulomas in the affected tissues, which is considered beneficial to the host and an important defense mechanism required to confine and control the infection. The cellular defense mechanisms in the host, which drive granuloma formation in tuberculosis, contribute to both clearance of the infection and tissue destruction. Healing occurs with progressive destruction of the parenchyma and variable degrees of fibrotic response [3]. In this context, lung remodelling in tuberculosis refers to anatomical and structural changes that are not easily reversed (laying down of extracellular matrix), in contrast to reversible changes, such as edema and cellular infiltration. Immune response to infection contributes to residual cavitation, lung fibrosis or scarring, and distortion of lung architecture, leading to volume loss and bronchiectasis [4].

Despite chemotherapy, lung remodelling in tuberculosis may result in variable degrees and patterns of lung functional impairment with significant morbidity [5–7]. In fact, treated pulmonary tuberculosis is considered one of the non-traditional risk factors for Chronic obstructive pulmonary disease (COPD) [8], and in tuberculosis endemic areas, cured pulmonary tuberculosis contributes to the prevalence of COPD as defined by Global Initiative for Chronic Obstructive Lung Disease (GOLD) criteria [9,10]. In addition, two population-based surveys from Latin American middle-aged and older adults demonstrated that tuberculosis is associated with airflow obstruction [11,12]. 

.We hypothesized that the degree of radiographic abnormalities, as detected by chest radiography, is associated with spirometric values: forced vital capacity (FVC) and forced expiratory volume in one second (FEV_1_). 

The aim of this study was to evaluate a novel scoring system based on the degree of chest radiographic abnormalities and the related spirometric values in patients with cured pulmonary tuberculosis. 

## Materials and Methods

The study was approved by the institutional Ethical and Research Review Board of the “Instituto Nacional de Enfermedades Respiratorias”, and written informed consent was obtained from each participant, and confidentially was ensured.

### Design, study setting, and population

This is a cross-sectional study. Patients were enrolled for a 30-month period, from 2006 to 2008, at a respiratory disease-dedicated Tuberculosis Clinic affiliated with a tertiary-care hospital of Mexico City, which mainly receives patients from the metropolitan area of Mexico City and neighboring states. All patients with pulmonary tuberculosis, who had completed antituberculosis treatment and whose cure was confirmed by negative sputum bacilloscopy and/or *Mycobacterium tuberculosis* culture, and who consented in writing to participate in the study, were eligible for inclusion. Excluded patients included those who were too physically impaired to perform the spirometry, active smokers, ex-smokers who had stopped smoking less than 6 months prior to being considered for the study, individuals exposed to smoke from biomass fuels, or had a history of occupational exposure to industrial fumes, patients with pleural tuberculosis or with any associated comorbidity that may have caused previous lung structural damage, or with functional limitation such as: interstitial lung disease, asthma, COPD, etc.

Patients were treated according to guidelines of the guidelines of Mexico’s National Tuberculosis Control Program [13]. 

### Study protocol, variables, and instruments of measurement

At recruitment, information on general characteristics, comorbidities, smoking history, and respiratory symptoms was obtained. Tuberculosis history and details of data on tuberculosis treatment and microbiological status were obtained from clinical charts. The degree of dyspnea was assessed according to the Medical Research Council (MRC) dyspnea scale [14]. 

### Pulmonary function testing

Spirometry, as well as pre- and post-bronchodilation, was performed in our pulmonary function laboratory using standard procedures for grading quality of the test and its interpretation [15–17]. 

Spirometry was performed with a PB 100 spirometer (Puritan Bennett, Lenexa, KS, USA) and EasyOne Plus Diagnostic Spirometry System SN: 46563/2002. The subject (seated and wearing nose clips) performed a forced exhalation, which yielded the forced expiratory volume in the first second (FEV_1_), peak expiratory flow (PEF), and forced vital capacity (FVC). Each subject was allowed to perform up to 15 forced expiratory maneuvers, in order to obtain three acceptable maneuvers with the highest FEV_1_ and FVC values reproducible within 150 mL. All tests were administered by specifically trained personnel.

The spirometry data were classified according to acceptability and reproducibility in a quality control code from A to F as described in Table 1 [18]. 

**Table 1:** Classification of spirometry data according to acceptability and reproducibility.

**Quality control code**	**Number of acceptable maneuvers**	**Difference between the two largest FEV_1_ or FVC measurements**	**Quality interpretation**
A	3	<150 mL	Very acceptable and very reproducible
B	3	<200 mL	Acceptable and reproducible
C	2	<200 mL	Less acceptable and reproducible
D	2	>200 mL	Less acceptable and variable
E	1		Inadequate
F	0		Inadequate

 The following values were recorded: FEV_1_, FVC, and their ratio (FEV_1_/FVC). Salbutamol (200 µg) was administered from a calibrated dose inhaler, and spirometry was repeated after 15 minutes. A significant bronchodilator response was defined as an increase of 200 mL and 12% in either the post-bronchodilator FEV_1_ or FVC, respectively. 

All measurements were expressed as both percentage of predicted normal (% predicted) and absolute values. The set of reference equations used to calculate the % predicted for spirometry values were obtained from Pérez-Padilla [19]. We used the lower limit of normal to interpret spirometric patterns, as suggested by the ERS/ATS guidelines, and the degree of spirometry impairment definition was based on the % predicted of FEV_1_ in: mild, >70%; moderate, 60–69%; moderately severe, 50–59%; severe, 35–49%; and very severe, <35% [16,17]. 

Oxygen pulse saturation (SpO_2_) was measured at rest using a pulse oximeter (Ohmeda Biox II 3740 Pulse Oximeter, Boulder, CO, USA).

### Development of the scoring system for grading radiographic abnormalities

**Figure 1:**
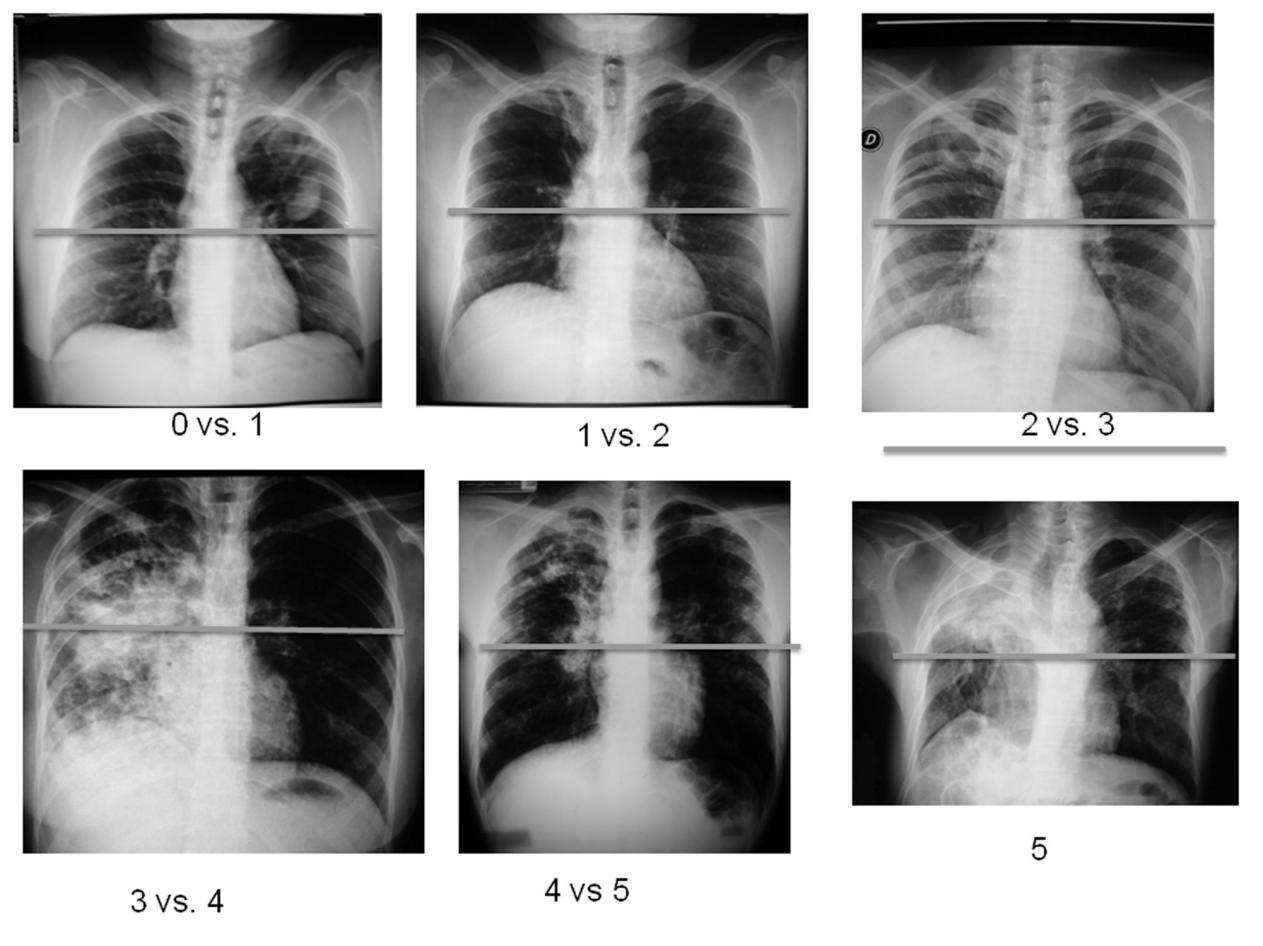
Representative example radiographs of quadrants for each score, according to the degree of radiographic abnormality using the same quadrant (upper left). The radiographs are from different patients from the study.

**Figure 2:**
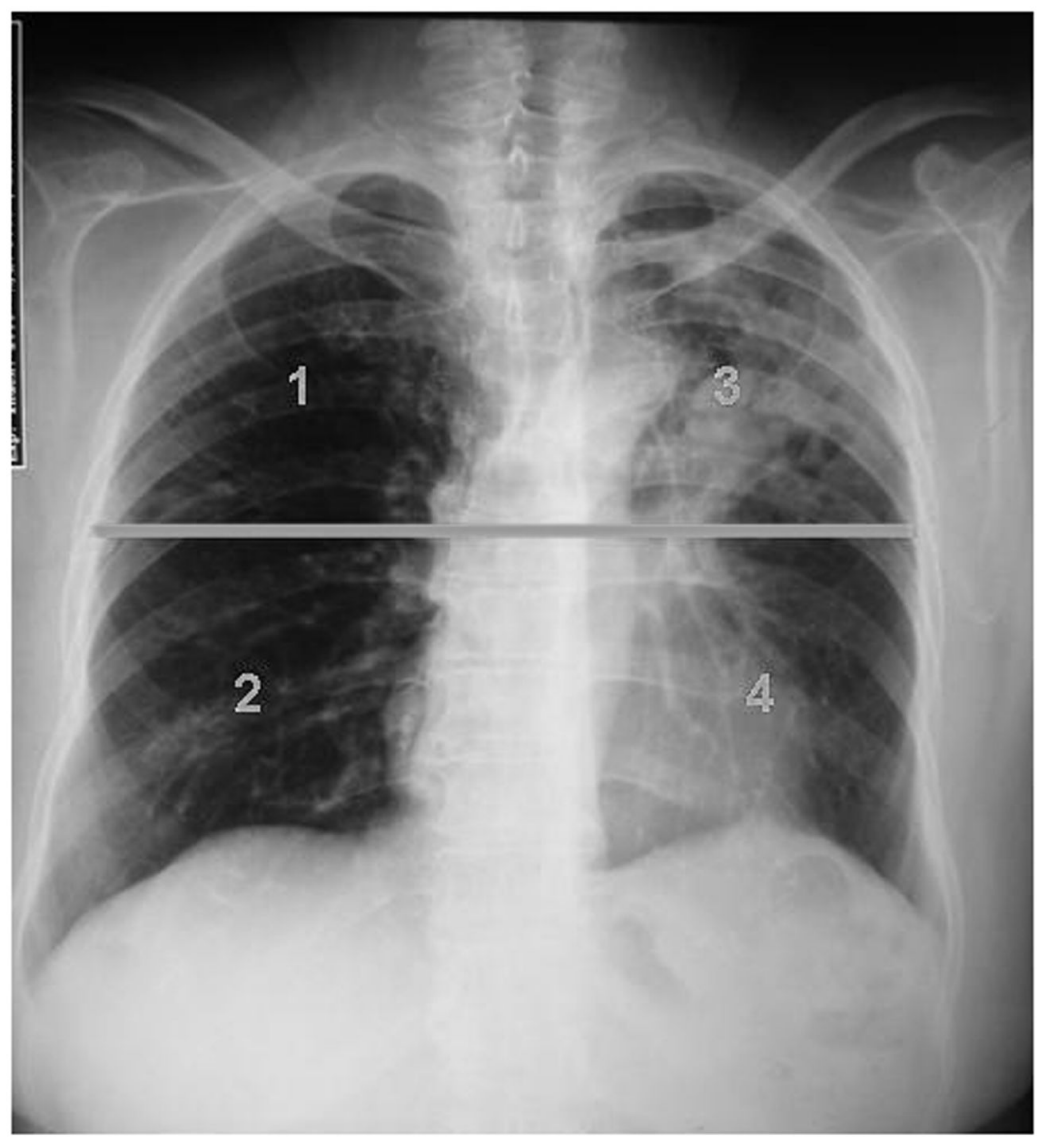
Representative example of the result of the scoring system for radiographic abnormalities. Score =6.5, which represents the mean value of two evaluations.

We used routine film posterior-anterior chest radiographs as a non-invasive technique to quantify the extent of lung remodelling in our patients. Each chest radiograph was assessed for the presence, distribution, and extent of pulmonary abnormalities, such as airspace consolidation and fibrosis, lung distortion, traction bronchiectasis, irregular interfaces, and parenchymal bands. We developed a quantitative scale to measure the degree of radiographic abnormalities. The pulmonary parenchyma was evaluated in four quadrants, with the division between the upper and lower lung in both sides being arbitrarily set at the carina section. Each quadrant was scored from 0 to 5, where 0 indicated a normal appearance, and 5 indicated severe abnormality. The score represented the percentage of lung parenchyma involvement. The maximum score for the four lung zones was 20 (Figures 1 and 2). The same image was read twice separately by two experienced observers (pulmonologist researcher RBS, reader one; and radiologist RCP, reader two) who were blinded to clinical or lung functional information. The time elapsed between the first and second measurements was two weeks. 

### Medical Research Council (MRC) Dyspnea Scale

The MRC dyspnea scale was used to identify patients according to their level of perceived breathlessness. We used the revised 5-grade version of the MRC dyspnea scale [14]. 

### Statistical Analysis

We reported the means and standard deviations (SD) of normally distributed values, and the medians and interquartile ranges (IQR) of non-normally distributed values. Categorical variables were summarized using absolute numbers and percentages. 

We measured the intra-observer and inter-observer concordance using the intra-class correlation coefficient (ICC) to evaluate the reliability and reproducibility of chest radiograph scoring. The Bland-Altman analysis [20] with 95% limit of agreement and Pitman´s test of difference in variance was used to test the intra-observer and inter-observer agreement for scoring of radiographic abnormalities for the first and second measurements.

The reported score for association analysis is the mean of four measurements.

To study the relation of radiographic abnormality scores to spirometric values (FVC and FEV_1_), Pearson’s correlation test was applied. Simple linear regression was used to quantify unadjusted associations between spirometric values (FVC and FEV_1_) and our scoring of radiographic abnormalities, age, gender, height (as a continuous variable) years of education (as a continuous variable), smoking status (former vs. never), pack-years of smoking (as a continuous variable), comorbidity, time of the disease (as a continuous variable), degree of dyspnea, and oxygen pulse saturation (as a continuous variable). All of the individuals in our study population are mestizos (as is most of the Mexican population); therefore, we did not adjust for race/ethnicity. 

Multiple linear regression was used to assess the cross-sectional relationship between our radiographic abnormality scoring system and spirometric values (FVC and FEV_1_) as the response variable. A forward selection model with p-value entry criterion of 0.05 was used to create adjusted models, using the following covariates: age, gender, height, smoking status (former vs. never), pack-years of smoking (as a continuous variable), degree of dyspnea, and oxygen pulse saturation (as a continuous variable). Factors for which there were statistically significant associations on adjusted models were chosen as covariates in subsequent adjusted models.

We examined models separately for absolute values and % predicted FVC and FEV_1_ values. Multicollinearity was a minor issue for most variables; most variance inflation factors (VIF) were smaller than 3.07. Assumptions of linearity, homoscedasticity, and normally distributed error terms were met for the sample. 

All analyses were performed with statistics software (Stata 12.0, StataCorp, College Station, TX, USA).

## Results

One hundred and twenty seven subjects were enrolled in the study. Participants were mostly middle-aged women with up to 10 years of formal schooling. One-third of the population was ex-smokers. The median (IQR) of pack-years of smoking was 1.65 (0.3–5). Almost one-half of the participants had underlying medical conditions, including diabetes (33.07%, n =42), hypertension (6.30%, n =8), HIV infection (2.36%, n =3), and hepatic cirrhosis (0.79%, n =1). Most participants (85%) reported having suffered only one episode of tuberculosis. Median (IQR) time elapsed from the end of anti-tuberculosis treatment to inclusion in the study was 11 (6–18) months. Fifty seven percent (n =73) of participants reported some degree of dyspnea. MRC dyspnea grades 1 and 2 were reported most often, in 39% (n =49) and 14% (n =18) cases, respectively (Table 2).

**Table 2:** Clinical characteristics of study participants.

**Variables**	**Total population**
Age (Years)^a^	48(32–64)
Gender, n (%)	
Male	55 (43%)
Female	72 (57%)
Education (Years of study)^a^	6 (4–10)
Smoking status, (former vs. never) n (%)	36 (28%)
Pack-years of smoking^a^	1.5 (0.3–5)
Comorbidity n (%)	54 (43%)
New case n (%)	108 (85%)
Time of the disease (days)^a*^ n = 77	90 (60–180) (4–720)
Time elapsed between the end of treatment and inclusion in the study (months)	
0 months n (%)	14 (11%)
1–12 months n (%)	51 (40%)
13–24 months n (%)	52 (41%)
27–252 months n (%)	10 (8%)
MRC Dyspnea Scale n (%)	
MRC 0	54 (42%)
MRC 1	49(39%)
MRC 2	18 (14%)
MRC 3	5(4%)
MRC 4	1 (1%)
Score of radiographic abnormalities^b^	6.6(4.14)

^a^ Median [interquartile range (IQR)], ^b^ Mean [Standard deviation (SD)] of four measurements

* Defined as the time elapsed between the onset of the symptoms and diagnosis of tuberculosis.

Of the 127 patients, 96.85% (n =123) exhibited some degree of radiographic abnormalities: 59% (n =75) exhibited a normal spirometric pattern, 24% (n =30) showed an obstructive spirometric pattern, and 17% (n =22) exhibited a restrictive spirometric pattern. In relation to the quality of the spirometry data, 67% (n = 85), 18% (n = 23), 12% (n = 15), and 3% (n = 4) were of control codes A through D, respectively. A positive bronchodilator response was observed in 12% (n =15) of patients, and 17% (n =21) demonstrated oxygen pulse saturation levels below 90% (Table 3).

**Table 3:** Spirometric results of the participants.

**Variables**	**Total population (n = 127**)
FEV_1_^a^	2.14 (0.80)
FEV_1_% pred^a^	83.33 (24.06)
FVC^a^	2.84 (0.94)
FVC% pred^a^	90.80 (23.90)
FEV_1_:FVC ratio (%)^a^	79.14 (13.17)
Normal spirometric pattern^a^, n (%)	75 (59%)
Obstructive spirometric pattern^a,^, n (%)	30 (24%)
Severity of spirometric abnormality based on the FEV_1_^b^	
Mild, n(%)	16 (13%)
Moderate, n (%)	4 (3%)
Moderately severe, n (%)	6 (5%)
Severe, n (%)	2 (1.5%)
Very severe, n (%)	2 (1.5%)
Restrictive spirometric pattern^a^, n (%)	22 (17%)
Response after use of bronchodilator, n (%)	15 (12%)
Oxygen pulse saturation	93 (91–95)
Oxygen desaturation (SpO2 <90%) n (%)	22 (17%)

Continuous data are expressed as mean [Standard deviation (SD)].

a Spirometry results are pre-bronchodilator.

b Severity of spirometric abnormality based on the predicted % FEV_1_ is as follows:

Mild >70, moderate 60–69, moderately severe 50–59, severe 35–49 and very severe <35 [15]

FEV_1_= forced expiratory volume in 1 second

FVC= forced vital capacity

% pred = percentage of predicted value

In 77 subjects, the time span between the start of disease symptoms and the moment of tuberculosis diagnosis was evaluated. The median (IQR) was significantly larger in the group with abnormal spirometric patterns than in the group with normal spirometric patterns: 165 (75–360) and 90 (30–180) days, respectively (p < 0.05).

The degree of radiographic abnormality median score (SD) reported by reader 1 (RBS) for the first evaluation was 6.39 (4.36); for the second, the median score (SD) was 5.52 (4.02). The ICC for both evaluations was 0.81 (CI: 95%, 0.67–0.95; p <0.001). The degree of radiographic abnormality median scores (SD) reported by reader 2 (RCP) were 6.75 (4.11) and 7.30 (4.54) for the first and second evaluations, respectively; the ICC for both evaluations was 0.78 (CI: 95%, 0.65–0.92; p <0.001). Inter-observer reproducibility for the first and second measurements showed an ICC of 0.83 (CI: 95%, 0.71–0.95; p <0.001) and 0.74 (CI: 95%, 0.58–0.90; p <0.001). 

The mean (SD) of the four radiographic abnormalities scores was 6.46 (4.14). 

### Bland-Altman Plot analysis

**Figure 3:**
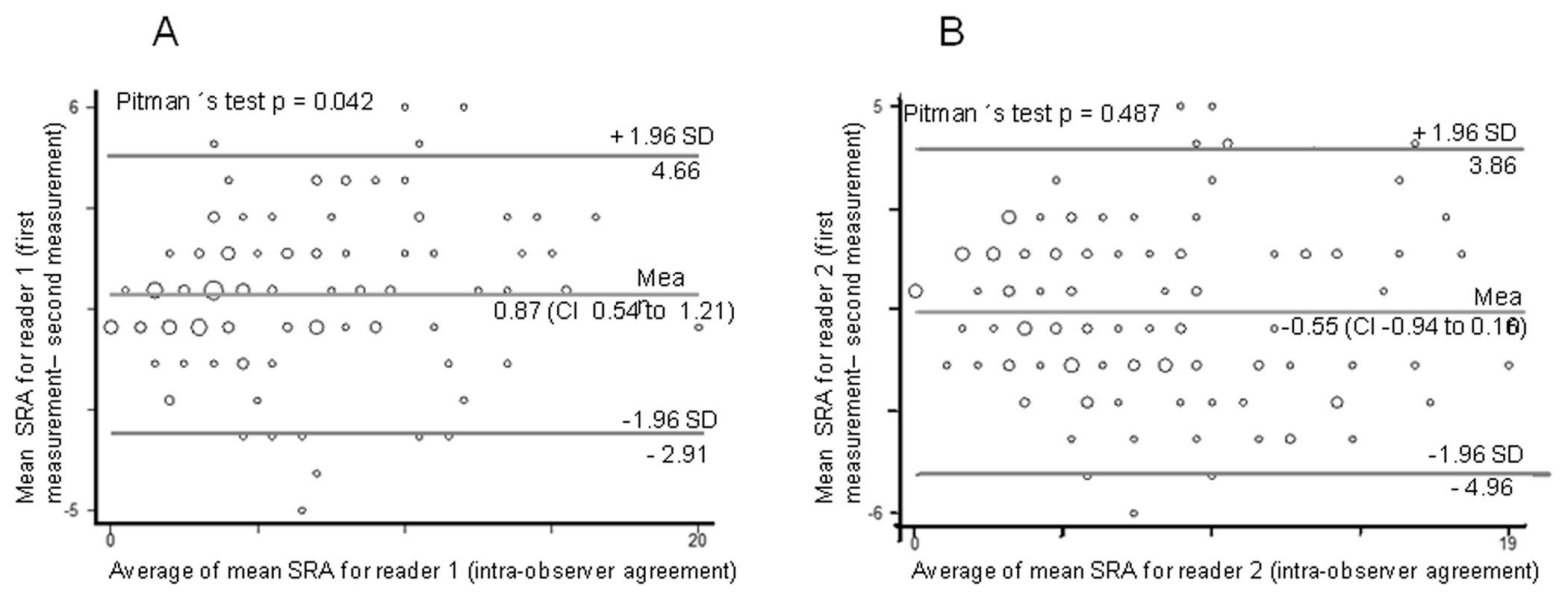
Bland-Altman plot of measurement differences against measurements average with a 95% limit of agreement superimposed for pair-wise comparisons of scoring of radiographic abnormalities (SRA), for intra-observer agreement: A) between reader one for the first and second measurements, B) between reader two for the first and second measurements.

The intra-observer variability of radiographic abnormality scoring by reader 1 (RBS) showed a mean bias of 0.87%, indicating a minor average systematic variability (Pitman´s test; p <0.05). The intra-observer variability for reader 2 (RCP) showed a minimal mean bias of -0.55% and no significant differences between the two evaluations (Pitman´s test; p >0.05), which indicates high agreement (Figure 3, A and B).

**Figure 4:**
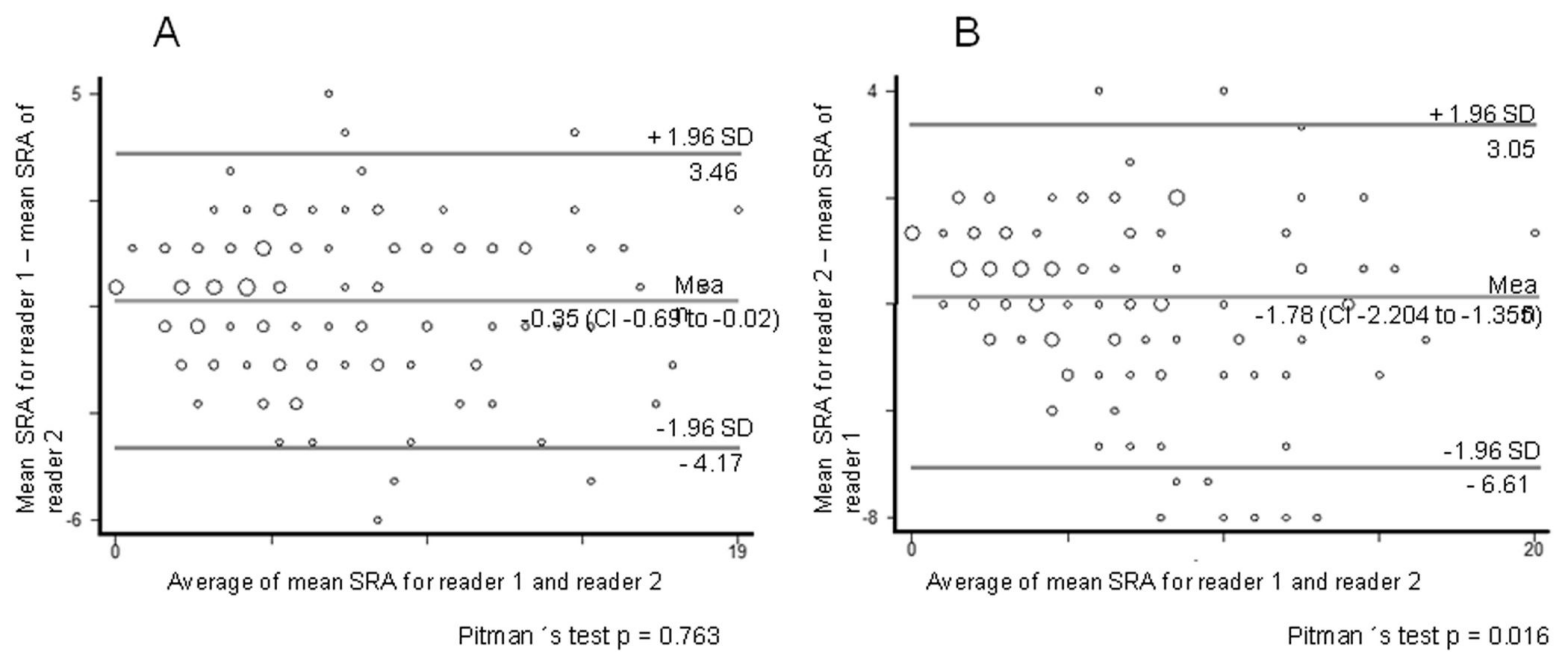
Bland-Altman plot of measurement differences against measurements averages with 95% limits of agreement superimposed for pair-wise comparisons of Scoring of radiographic abnormalities (SRA), for inter-observer agreement: A) between reader 1 vs reader 2 for the first measurement, B) between reader one vs reader two for the second measurement.

A comparison of radiographic abnormality scores assessed between reader 1 and reader 2 for the first measurement showed a minimal difference (- 0.35%) and a high agreement between the two readers; Pitman´s test showed that there is no significant (p >0.05) difference between the measuring errors by the two readers. In contrast, the results for the second measurement showed moderate agreement (mean bias of -1.78%) and significant variation between the two readers (Pitman´s test; p <0.05) (Figure 4, A and B).

**Figure 5:**
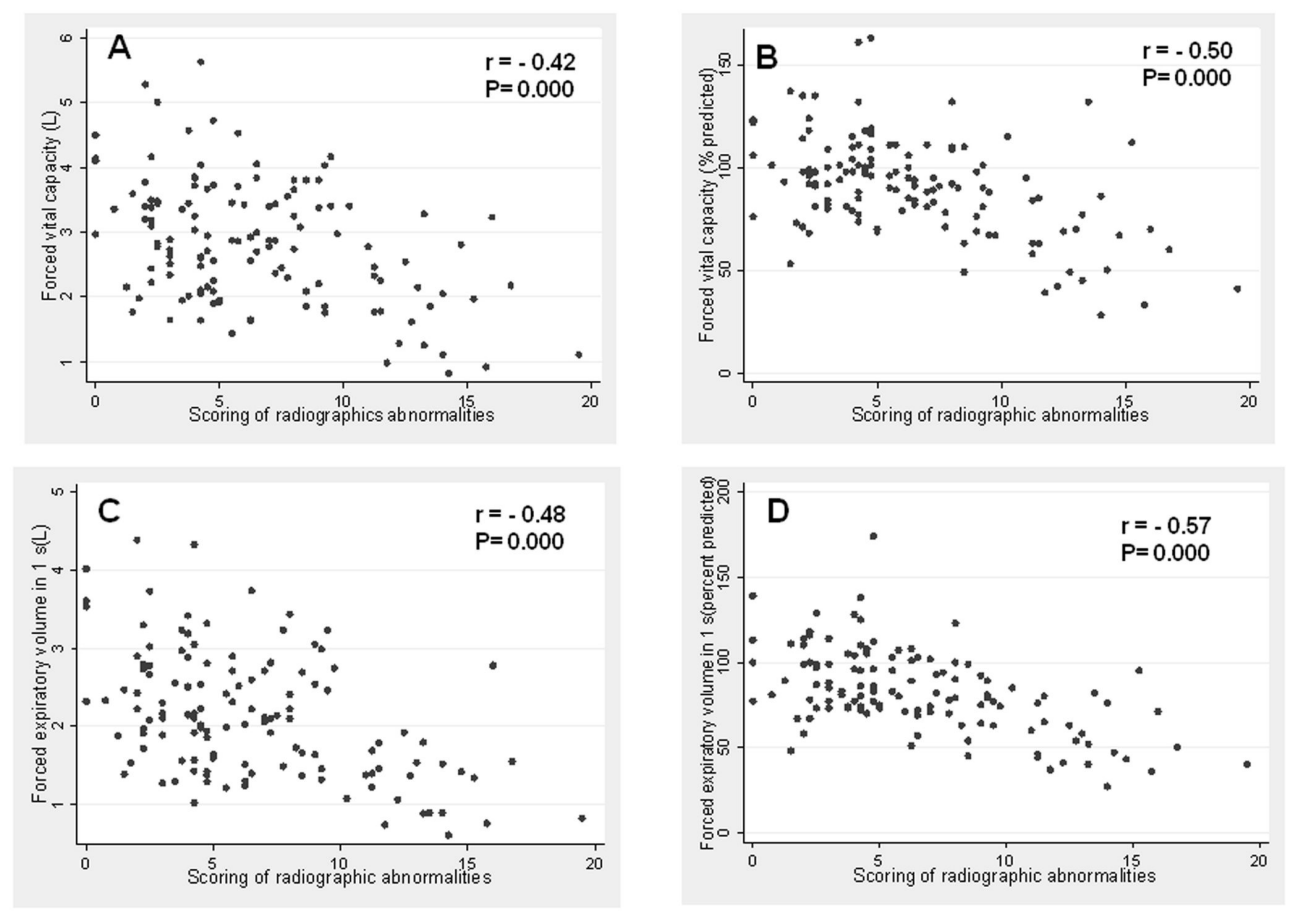
Association of absolute values and percentage of predicted of forced vital capacity (FVC) (A, B); for whom forced expiratory volume in 1 s (FEV_1_) (C, D) with the score of degree of radiographic abnormalities. (FEV_1_).

 Pearson’s correlation test demonstrated a significant negative correlation between absolute and % predicted normal values of FVC and FEV_1_ (L) and the scoring of radiographic abnormalities (Figure 5). 

Simple linear regression analysis showed that scoring of radiographic abnormalities, age, gender and MRC dyspnea scale were negatively associated with absolute values of FVC, while height, smoking status (former vs. never), pack-years of smoking and oxygen pulse saturation were positively associated with absolute values of FVC (Tables 4 and 5). The association with % predicted normal values of FVC showed a negative association with SRA and dyspnea MRC3, while oxygen pulse saturation was positively associated. The same analysis for FEV_1_ revealed that SRA and dyspnea MRC3 were negatively associated, whereas oxygen pulse saturation was positively associated (Tables 6 and7).

**Table 4:** Parameter estimates from simple linear regression analysis of absolute values of forced vital capacity (FVC) in relation to the scoring of radiographic abnormalities and other factors.

	**FVC (L)**		
Variables	Parameter estimates (95% CI)	P value	Adjusted R-squared
Scoring of radiographic abnormalities	-0.10 (-0.13 to -0.06)	0.000	0.1584
Age	-0.02 (-0.03 to -0.01)	0.000	0.0968
Gender	-0.88 (-1.18 to -0.58)	0.000	0.2086
Height	0.07 (0.05 to 0.08)	0.000	0.3417
Education (years of study)	0.07 (0.04 to 0.11)	0.000	0.1215
Smoking status (former vs. never)	0.67 (0.323 to 1.022)	0.000	0.0968
Pack-years of smoking	0.03 (-0.09 to 0.15)	0.000	-0.0207
Comorbidity	-0.08 (-0.23 to 0.06)	0.246	0.0028
Time of the disease (days)	-0.00 (-0.00 to 0.01)	0.177	0.0112
MRC Dyspnea Scale			
MRC 1	-0.33 (-0.66 to -0.00)	0.046	0.2091
MRC 2	-0.12 (-1.57 to -0.66)	0.000	
MRC 3	-1.35 (-2.13 to -0.58)	0.001	
MRC 4	-2.37 (-4.04 to -0.69)	0.006	
Oxygen pulse saturation	0.08 (0.04 to 0.12)	0.000	0.1013

**Table 5:** Parameter estimates from simple linear regression analysis of absolute values of forced expiratory volume in 1 second (FEV_1_) in relation to the scoring of radiographic abnormalities and other factors.

	**FEV_1_ (L)**		
**Variables**	**Parameter estimates (95% CI)**	**P value**	**Adjusted R-squared**
Scoring of radiographic abnormalities	-0.09 (-0.12 to -0.06)	0.000	0.22
Age	-0.02 (-0.03 to -0.01)	0.000	0.1567
Gender	-0.59 (-0.86 to -0.33)	0.000	0.1291
Height	0.05 (0.04 to 0.07)	0.000	0.2886
Education (years of study)	0.07 (0.04 to 0.09)	0.000	0.1503
Smoking status (former vs. never)	0.48 (0.18 to 0.78)	0.002	0.0656
Pack-years of smoking	0.00 (-0.09 to 0.09)	0.963	-0.0293
Comorbidity	-0.08 (-0.20 to 0.04)	0.215	0.215
Time of the disease (days)	-0.00 (-0.00 to 0.00)	0.073	0.0293
MRC Dyspnea Scale			
MRC 1	-0.34 (-0.62 to -0.06)	0.018	0.2029
MRC 2	-0.94 (-1.32 to -0.55)	0.000	
MRC 3	-1.17 (-1.84 to -0.51)	0.001	
MRC 4	-1.85 (-3.28 to -0.43)	0.011	
Oxygen pulse saturation	0.07 (0.04 to 0.11)	0.000	0.1279

**Table 6:** Parameter estimates from simple linear regression analysis of percent (%) predicted values of forced vital capacity (FVC) in relation to the scoring of radiographic abnormalities and other factors.

	**Percent predicted of FVC (%)**		
**Variables**	**Parameter estimates (95% CI)**	**P value**	**Adjusted R-squared**
Scoring of radiographic abnormalities	-2.90 (-3.79 to -2.02)	0.000	0.25
Education (years of study)	-0.63 (-1.56 to 0.31)	0.186	0.00
Smoking status (former vs. never)	1.850 (-7.50 to 11.20)	0.696	-0.01
Pack-years of smoking	-0.38 (-2.75 to 1.99)	0.748	-0.03
Comorbidity	2.57 (-1.04 to 6.18)	0.161	0.01
Time of the disease (days)	-0.03 (-0.07 to 0.01)	0.162	0.01
MRC Dyspnea Scale			
MRC 1	-2.85 (-11.68 to 5.98)	0.524	0.1053
MRC 2	-6.78 (-18.96 to 5.40)	0.273	
MRC 3	-41.13 (-62.06 to -20.21)	0.000	
MRC 4	-44.83 (-90 to 0.34)	0.052	
Oxygen pulse saturation	1.18 (0.14 to 2.22)	0.026	0.0312

**Table 7:** Parameter estimates from simple linear regression analysis of percent (%) predicted values of forced expiratory volume in 1 second (FEV_1_) in relation to the scoring of radiographic abnormalities and other factors.

**Variables**	**Parameter estimates (95% CI)**	**P value**	**Adjusted R-squared**
Scoring of radiographic abnormalities	-3.29 (-4.14 to -2.44)	0.000	0.31
Education (years of study)	-0.532 (-1.47 to 0.41)	0.263	0.0021
Smoking status (former vs. never)	2.83 (-6.57 to 12.24)	0.552	-0.0051
Pack-years of smoking	-1.183 (-3.59 to 1.23)	0.325	-0.0001
Comorbidity	3.29 (-0.33 to 6.90)	0.074	0.0174
Time of the disease (days)	-0.03 (-0.07 to 0.01)	0.090	0.0251
MRC Dyspnea Scale			
MRC 1	-4.48 (-13.44 to 4.48)	0.324	0.0921
MRC 2	-9.74 (-22.10 to 2.62)	0.121	
MRC 3	-38.90 (-60.12 to -17.67)	0.000	
MRC 4	-41.30 (-87.11 to 4.52)	0.077	
Oxygen pulse saturation	1.38 (0.34 to 2.42)	0.009	0.0451

Multiple linear regression models revealed that after adjusting for age, gender, height, smoking status, pack-years of smoking, degree of dyspnea, and oxygen pulse saturation, the degree of radiographic abnormalities was independently associated with absolute values of FVC (0.07 L decrease for each unit increase in score of lung damage; CI: 95%, -0.01 to -0.04; p <0.001); FEV_1_ (0.07 L decrease for each unit increase in score of lung damage; CI: 95%, -0.10 to -0.05; p <0.001); and with % predicted values of FVC (2.48% decrease for each unit increase in score of lung damage; CI: 95%, -3.45 to -1.50; p <0.001); and FEV_1_ [2.92% decrease for each unit increase in score of lung damage; CI: 95%, -3.87 to -1.97; p <0.001) (Tables 8 and 9). 

**Table 8:** Multiple linear regression models to estimate the relationship of absolute spirometric values (FVC and FEV_1_) with scoring of radiographic abnormalities among patients with cured pulmonary tuberculosis.

**Variables**	Model for FVC(L)*		Model for FEV_1_ (L)*	
	Parameter estimates (95% CI)	P value	Parameter estimates (95% CI)	P value
Scoring of radiographic abnormalities*	-0.07 (-0.01 to -0.04)	0.000	-0.07 (-0.10 to -0.05)	0.000
Age	-0.01 (-0.02 to -0.00)	0.009	-0.01 (-0.02 to -0.01)	0.002
Gender (Female)	-0.67 (-1.01 to -0.33)	0.000	-0.40 (-0.70 to -0.11)	0.008
Height	0.03 (0.01 to 0.04)	0.013	0.02 (0.01 to 0.04)	0.006
Smoking status (former vs. never)	0.19 (-0.12 to 0.50)	0.227	0.12 (-0.15 to 0.39)	0.385
Pack-years of smoking	0.00 (-0.07 to 0.07)	0.065	0.01 (-0.07 to 0.04)	0.638
MRC Dyspnea Scale				
MRC 0	Reference		Reference	
MRC 1	-0.13 (-0.35 to 0.10)	0.262	-0.17 (-0.36 to 0.03)	0.094
MRC 2	-0.61 (-0.95 to -0.28)	0.000	-0.44 (-0.73 to -0.15)	0.004
MRC 3	-0.96 (-1.60 to -0.33)	0.003	-0.58 (-1.14 to -0.03)	0.040
MRC 4	-0.48 (-1.69 to -0.73)	0.431	-0.06 (-1.12 to 1.00)	0.918
Oxygen pulse saturation	0.02 (-0.01 to 0.05)	0.138	0.03 (0.00 to 0.05)	0.039
Adjusted R^2^	0.64		0.62	

* Multivariate linear regression model.

CI: Confidence interval

**Table 9:** Multiple Linear Regression Models to Estimate the Relationship of percent (%) predicted spirometric values (FVC and FEV_1_) with scoring of radiographic abnormalities among patients with cured pulmonary tuberculosis.

**Variables**	Model for percent predicted FVC*		Model for percent predicted FEV_1_*	
	Parameter estimates (95% CI)	P value	Parameter estimates (95% CI)	P value
Scoring of radiographic abnormalities*	-2.48 (-3.45 to -1.50)	0.000	-2.92 (-3.87 to -1.97)	0.000
Smoking status (former vs never)	-0.27 (-11.28 to 10.74)	0.961	1.82 (-8.82 to 12.46)	0.735
Pack-years of smoking	-0.31(-2.76 to 2.14)	0.804	-0.98 (-3.34 to 1.40)	0.417
MRC Dyspnea Scale				
MRC 0	Reference		Reference	
MRC 1	-1.16 (-9.35 to 7.03)	0.517	-2.39 (-10.30 to 5.53)	0.551
MRC 2	-2.42 (-14.36 to 9.52)	0.689	-3.92 (-15.45 to -7.62)	0.503
MRC 3	-23.84 (-46.09 to 0.88)	0.042	-16.10 (-38.29 to 6.08)	0.153
MRC 4	-24.18 (-68.32 to 19.96)	0.280	-13.29 (-55.94 to 29.36)	0.538
Oxigen pulse saturation	0.04 (-1.14 to 1.06)	0.943	0.26 (0.80 to 1.33)	0.619
Adjusted R^2^	0.24		0.30	

* Multivariate linear regression model.

CI: Confidence interval

Additional adjustment for smoking status (former vs. never), pack-years of smoking, height, and age changed parameter estimates by less than 3% for absolute and % predicted of FVC and FEV_1_ values, suggesting that those variables were not confounders (Tables 10 and 11). 

**Table 10:** Additional multiple linear regression analysis to evaluate confusion between the relationship of absolute values of forced vital capacity (FVC) (L) and forced expiratory volume in 1 second, FEV1 (L) with scoring of radiographic abnormalities.

	**Absolute values**	
	**FVC (L)**	**FEV_1_ (L)**
	Parameter estimates (95% CI)	Parameter estimates (95% CI)
SRA^a^	-0.071 (-0.098 to -0.044)	-0.073 (-0.096 to -0.049)
SRA^b^	-0.071(-0.098 to -0.043)	-0.072 (-0.097 to -0.048)
Percent change for age*	0	-1.37
SRA^c^	-0.074 (-0.101 to -0.046)	-0.075 (-0.099 to -0.051)
Percent change for height*	4.22	2.74
SRA^d^	-0.075 (-0.101 to -0.048)	-0.075 (-0.098 to 0.052)
Percent change for smoking status (former vs. never)	5.63	2.74
SRA^e^	-0.071 (-0.098 to -0.045)	-0.073 (-0.096 to - 0.050)
Percent change for pack-years of smoking*	0	0

CI; Confidence Interval, SRA Scoring of radiographic abnormalities, * Percent change >10% means confusion.

a Adjusted for age, gender, height, smoking status (former vs. never), pack-years of smoking, MRC dyspnea scale and oxygen pulse saturation.

b Adjusted for gender, height, smoking status (former vs. never), pack-years of smoking, MRC dyspnea scale and oxygen pulse saturation.

c Adjusted for age, gender, smoking status (former vs. never), pack-years of smoking, MRC dyspnea scale and oxygen pulse saturation.

d Adjusted for age, gender, height, pack-years of smoking, MRC dyspnea scale and oxygen pulse saturation.

e Adjusted for age, gender, height, smoking status (former vs. never), MRC dyspnea scale and oxigen pulse saturation.

**Table 11:** Additional multiple linear regression analysis to evaluate confusion between the relationship of percent predicted values of forced vital capacity (%FVC) and forced expiratory volume in 1 second, (%FEV_1_) with scoring of radiographic abnormalities.

	**% predicted FVC**	**% predicted FEV_1_**
	**Parameter estimates (95% CI)**	**Parameter estimates** (**95% CI**)
SRA^a^	-2.48 (-3.45 to -1.50)	-2.92 (-3.87 to -1.97)
SRA^b^	-2.47 (-3.43 to -1.51)	-2.95 (-3.88 to -2.02
Percent change for smoking status (former vs. never)*	-0.40	1.03
SRA^c^	-2.49 (-3.46 to -1.52)	-2.96 (-3.90 to-2.02
Percent change for pack-years of smoking*	0.40	1.37

CI; Confidence Interval, SRA Scoring of radiographic abnormalities, * Percent change >10% means confusion.

aAdjusted for smoking status (former vs. never), pack-years of smoking, MRC dyspnea scale, and oxygen pulse saturation.

b Adjusted for pack-years of smoking, MRC dyspnea scale, and oxygen pulse saturation.

c Adjusted for smoking (former vs. never), MRC dyspnea scale, and oxygen pulse saturation.

None of the interactions between age and the scoring of radiographic abnormalities were significant.

## Discussion

Our results indicate that after adjustment for age, height, smoking status (former vs. never), pack-years of smoking, and degree of dyspnea, the scoring degree of radiographic abnormalities was significantly and inversely associated with FVC and FEV_1_, in the studied patients with cured pulmonary tuberculosis. This association was independent of the reader, as evidenced by the good reproducibility of the data, and that intra-observer and inter-observer agreement of the SRA varied from good to excellent.

 Moreover, our findings confirm that radiographic abnormalities, lung functional impairment, and dyspnea are common in patients from our institution who have successfully completed tuberculosis treatment. We demonstrated that 96.85% (123/127) of patients had some degree of radiographic abnormality, 41% (52/127) had impaired lung function, and 58% (73/127) had some degree of dyspnea as well.

 While our study is cross-sectional, the demonstrated associations are in keeping with previous research, which indicates that cured pulmonary tuberculosis causes variable degrees and patterns of lung functional impairment [5,6,21]. A correlation between lung structure— evaluated by conventional computerized tomography—and lung function in pulmonary tuberculosis has also been demonstrated [22].

In addition, Plit and colleagues [23] reported that although antituberculosis treatment improved lung function in patients with pulmonary tuberculosis, a large proportion of patients experience residual impairment: 28% develop airflow limitation and 24% develop a restrictive pattern. They also demonstrated that lung damage—evaluated through radiographic scores—is a factor that influences post-treatment lung function. 

Normal spirometric patterns, observed in 59% (n =75) of participants, were the most prevalent in our study. Among the patients with spirometric impairment, an obstructive pattern was the most prevalent, as observed in 57.69% (n =30/52) of cases, followed by a restrictive pattern in 42.30% (n = 22/52) of cases. This contrasts with another study, in which mixed ventilatory disorder was the most prevalent (34%), followed by obstructive (24%) and restrictive (18%) patterns, and in which only 24% of the sample showed a normal spirometry pattern [24]. 

Long delays in diagnosis and treatment occur in Mexico, further aggravating not only the transmission of the disease, but also the possibility of increased lung damage, which, according to our results, seems to affect lung function negatively. We demonstrated that the median (IQR) time span between the onset of disease symptoms and tuberculosis diagnosis was significantly longer in patients with lung functional impairment than in patients without lung functional impairment. Our results contrast with a recent publication by Vecino et al., who showed that pulmonary impairment after tuberculosis is not related to the delay in tuberculosis diagnosis or treatment and does not change significantly during follow-up [21].

Our findings are in agreement with previous reports, conducted in different settings, such as population-based studies, that demonstrated a strong association between pulmonary tuberculosis and subsequent impairment in lung function [25,26]. In previous studies, considerable variability in frequency and patterns of lung functional impairment among patients with pulmonary tuberculosis is most probably due to differences in patient characteristics, such as previous treatment schedule, smoking status, and varying intervals of treatment onset. 

We found a heterogeneous spectrum of radiographic abnormalities, including parenchymal fibrosis, bronchiectasis, and emphysema (data not shown). The mechanism of airway obstruction in our patients can be due to partial destruction inside the airways and/or of lung parenchyma, causing loss of radial traction and consequent narrowing of the airway. The restrictive pattern that we also observed may be due to alterations in lung parenchyma and/or pleural affection.

The mean of the differences between equations for all cases of the inter-observer and intra-observer evaluations of SRA ranged from -1.78 to 0.87, indicating a minor average systematic variability. The best agreement occurred between the readers for the first measurement of SRA, with a mean difference of -0.354 (Pitman´s test, p >0.05), and the higher variability occurred for the second measurement of SRA with a mean difference of -1.78 (Pitman´s test, p <0.05). 

Our results showed that the use of our scoring system for evaluating the degree of radiographic abnormality predicted lung functional impairment; in this context, measurement of lung functional impairment in subjects with cured pulmonary tuberculosis may be useful, in particular, in subjects with high degrees of radiographic abnormalities. 

It is also important to assess the degree and impact of breathlessness in the overall clinical evaluation of these patients. However, because these patients may reduce their activities, a simple assessment of dyspnea may be insufficient; hence, an assessment of functional status, utilizing a tool such as the MRC dyspnea scale, is necessary to fully evaluate the functional impact of radiographic abnormalities in cured pulmonary tuberculosis patients. This was demonstrated in a 6-month longitudinal study, developed in 115 patients with smear-positive pulmonary tuberculosis from Papua, Indonesia, whose permanent lung damage influenced exercise tolerance and quality of life in a region with a high number of pulmonary tuberculosis cases and chronic lung dysfunction [27].

From the operational point of view, our study had the advantage of using a low-cost strategy to evaluate lung function. We were able to document an association between the degree of radiographic abnormalities and spirometric values in a single visit conducted after treatment completion. Previous studies have used prospective designs or have evaluated lung damage by conventional computerized tomography [21,23]. Both methods entail problems with patient compliance and higher costs, which are particularly significant in low and medium resource settings. 

Despite our rather simple design, our results are similar to those shown in large-population based studies, prospective designs or studies that have used computerized tomography to evaluate lung damage [21,23–26].

### Strengths and limitations

Some strengths and limitations of this study must be addressed. To date, there are few studies that link radiographic abnormalities with pulmonary function in cured pulmonary tuberculosis. The use of ICCs and Bland-Altman plots method with limits of agreement for evaluation of our SRA provide a robust examination of agreement.

Our results were obtained from a selected population with cured pulmonary tuberculosis, therefore, we may assume that the lung functional impairment and dyspnea are due to effects of tuberculosis-induced lung remodelling. Nevertheless, due to its cross-sectional design, a potential weakness of this study is the lack of spirometry data prior to tuberculosis; hence, we cannot state with certainty that pulmonary tuberculosis caused the observed spirometry changes. or that there has been some damage from pulmonary tuberculosis leading to reduced spirometric results, but still leaving them defined as normal (FVC fell from 110% of predicted to 81% of predicted).

Our use of chest X-ray instead of computed tomography allowed us to reduce costs without affecting our ability to demonstrate a relation between radiographic abnormalities and spirometric values. Moreover, we were able to document that this low cost and frequently available resource is useful and valid to determine extent of damage to lung parenchyma. 

### Future research

Additional studies are needed to investigate pathophysiological factors associated with lung remodelling, changes in pulmonary function, exercise tolerance, and health-related quality of life, following pulmonary TB treatment. Understanding this relationship will be useful to assess the long-term impact of tuberculosis on patients’ quality of life and to implement preventive and control measures. 

## Conclusion

This study showed that spirometric values were associated to the extent of radiographic abnormalities assessed by chest radiography using a simple, valid, and reproducible scoring method, and its used in this setting appears acceptable. Intra-observer and inter-observer agreement of the SRA varied from good to excellent. In patients with cured pulmonary tuberculosis from this population spirometric impairment and dyspnea are common. 
